# 

**Published:** 1856-01

**Authors:** 


### CONTENTS.

### ORIGINAL COMMUNICATIONS.

PAGE.

ARTICLE I.—Remarks on the Pathology of Phlegmasia Doleus. By HAYDEN COE, M. D., Atlanta, Georgia........ 257

ARTICLE II.—Reports from the Case Book of THOS. S. POWELL, M. D., Sparta, Georgia........ 264

ARTICLE III.—Cholera. By HORATIO N. HOLLIFIELD, A. M., M. D., Sandersville, Ga........ 269

ARTICLE IV.—Remarkable Case of Tumor of the Side. By E. H. ROACH, M. D., Atlanta, Ga........ 272

### SELECTIONS.

On the use of Sulphate of Cinchonia as a substitute for the Sulphate of Quinia, 274

Glycogenia—The secretion of Sugar in the Human Economy........ 281

Case of Yellow Fever occurring in St. Louis, August, 1855........ 289

Observations on Curative Evidence........ 290

Illustrations of the Influence of Pregnancy........ 294

On the Rudimentary Reproduction of Extremities after their Spontaneous Amputation........ 296

On Intra-Uterine Goitre, or Bronchocele........ 298

Case in which Six Drachms of Arsenic were taken........ 305

Chemical Pathology........ 305

Editorial and Miscellaneous........ 306

